# Revisiting Non-Thermal Food Processing and Preservation Methods—Action Mechanisms, Pros and Cons: A Technological Update (2016–2021)

**DOI:** 10.3390/foods10061430

**Published:** 2021-06-20

**Authors:** James S. Chacha, Liyan Zhang, Chigozie E. Ofoedu, Rashid A. Suleiman, Joachim M. Dotto, Ume Roobab, Adedoyin O. Agunbiade, Haile Tesfaye Duguma, Beatha T. Mkojera, Sayed Mahdi Hossaini, Waheed A. Rasaq, Ivan Shorstkii, Charles Odilichukwu R. Okpala, Malgorzata Korzeniowska, Raquel P. F. Guiné

**Affiliations:** 1 Department of Food Technology, Nutrition, and Consumer Sciences, Sokoine University of Agriculture, P.O. Box 3006 Chuo Kikuu, Tanzania; rashid@sua.ac.tz (R.A.S.); beatha.mkojera@sua.ac.tz (B.T.M.); 2 School of Food Science and Engineering, South China University of Technology, Guangzhou 510640, China; liyanzh@scut.edu.cn (L.Z.); mahroba73@gmail.com (U.R.); adedoyinodebade073128@gmail.com (A.O.A.); hailetesfaye840@gmail.com (H.T.D.); 3 Department of Food Science and Technology, School of Engineering and Engineering Technology, Federal University of Technology, Owerri 460114, Nigeria; 4 School of Life Sciences and Bioengineering, Nelson Mandela African Institution of Science and Technology, P.O. Box 447 Arusha, Tanzania; j.dottoe@gmail.com; 5 Department of Food Technology, University of Ibadan, Ibadan 200284, Nigeria; 6 Department of Post-Harvest Management, College of Agriculture and Veterinary Medicine, Jimma University, P.O. Box 378 Jimma, Ethiopia; 7 DIL German Institute of Food Technologies, Prof.-von-Klitzing-Str. 7, D-49610 Quakenbrück, Germany; s.hossaini@dil-ev.de; 8 Department of Applied Bioeconomy, Wrocław University of Environmental and Life Sciences, 51-630 Wrocław, Poland; waheed.rasaq@upwr.edu.pl; 9 Department of Technological Equipment and Life-Support Systems, Kuban State Technological University, 350072 Krasnodar, Russia; i-shorstky@mail.ru; 10 Faculty of Biotechnology and Food Sciences, Wroclaw University of Environmental and Life Sciences, 51-630 Wrocław, Poland; malgorzata.korzeniowska@upwr.edu.pl; 11 CERNAS Research Centre, Polytechnic Institute of Viseu, 3504-510 Viseu, Portugal

**Keywords:** non-thermal food processing, agro-food industry, product development, food preservation, food processors

## Abstract

The push for non-thermal food processing methods has emerged due to the challenges associated with thermal food processing methods, for instance, high operational costs and alteration of food nutrient components. Non-thermal food processing involves methods where the food materials receive microbiological inactivation without or with little direct application of heat. Besides being well established in scientific literature, research into non-thermal food processing technologies are constantly on the rise as applied to a wide range of food products. Due to such remarkable progress by scientists and researchers, there is need for continuous synthesis of relevant scientific literature for the benefit of all actors in the agro-food value chain, most importantly the food processors, and to supplement existing information. This review, therefore, aimed to provide a technological update on some selected non-thermal food processing methods specifically focused on their operational mechanisms, their effectiveness in preserving various kinds of foods, as revealed by their pros (merits) and cons (demerits). Specifically, pulsed electric field, pulsed light, ultraviolet radiation, high-pressure processing, non-thermal (cold) plasma, ozone treatment, ionizing radiation, and ultrasound were considered. What defines these techniques, their ability to exhibit limited changes in the sensory attributes of food, retain the food nutrient contents, ensure food safety, extend shelf-life, and being eco-friendly were highlighted. Rationalizing the process mechanisms about these specific non-thermal technologies alongside consumer education can help raise awareness prior to any design considerations, improvement of cost-effectiveness, and scaling-up their capacity for industrial-level applications.

## 1. Introduction

One of the major purposes of food processing technologies, from the very early to its later types over the years, has been to guarantee the safety of foodstuffs, as well as prolong their shelf-life. However, a diversity of changes have taken place as the many food processing technologies advanced and evolved, especially those within the agro-food industry/sector [1,2,3,4]. Indeed, all have happened largely because of the evolving nature of consumer demands for foods that are fresh, nutritious and with increased shelf life [1,2,3,4]. Since consumers are the key players in the agro-food chain, their demands over the years have been a key push factor to other players in the agro-food chain, such as food technologists, in devising processes that ensure such demands are met.

For many years, the primary methods for treatments for the microbiological stabilization and the preservation of the sensory and nutritional properties of food products have largely been those associated with heat, which unfortunately has adversely affected both the nutrient contents and sensory characteristics of foods [5,6]. Although one of the key aims of the heat processing technologies has been to reduce food spoilage and pathogenic microorganisms, they have in many situations resulted in many undesirable and unwanted modifications in food texture, appearance, sensory aspects, nutrients content, and the concentration of thermo-sensitive bioactive compounds. Therefore, the destruction of food nutritional components and the consumer demand for foods with desirable sensory qualities have remained the two key drivers towards innovation of the non-destructive food technologies [4,7]. Such food technologies are designed in such a way that while they ensure microbial inactivation, the foods still retain their sensory aspects [8,9].

Non-thermal food processing simply refers to methods where the food materials receive microbiological inactivation without the direct application of heat [3,4,8]. Such technologies, largely combined with hurdle technology to replace those conventional thermal food processing ones, are increasingly viewed as either emerging, novel or new food processing methods [4,10]. Such novel technologies have included pulse electric fields (PEF), high-pressure processing (HPP) [6], ozone treatment [11,12,13], pulsed light, non-thermal plasma/cold plasma (NTP) and ultrasound technology [14,15]. The technologies can be grouped into two major groups: physical processes (pulse electric field, high pressure processing, ultraviolet radiation, pulsed light, ultrasound and ionizing radiation) and chemical processes (ozone treatment, and cold plasma).

**Figure 1:**
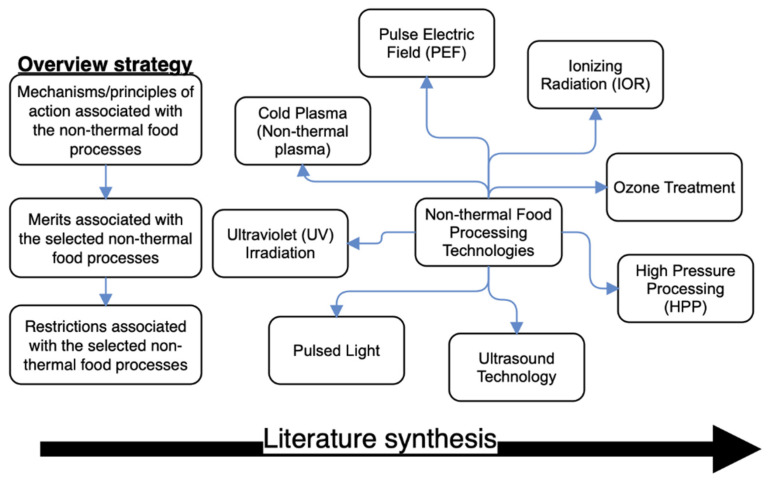
Schematic representation of non-thermal food processing technologies synthesized (which include: pulsed electric field (PEF), pulsed light (PL), ultraviolet radiation (UV), ionizing irradiation (IOR), high-pressure processing (HPP), ozone treatment, cold plasma (non-thermal plasma), and ultrasound technology) with the overview strategy (which include: mechanisms/principles of action, and the associated merits, and restrictions).

Besides being well established in scientific literature, research into non-thermal food processing technologies are constantly on the rise, as applied to a wide range of food products. Due to such remarkable progress by scientists and researchers, there is need for continuous synthesis of relevant scientific literature, for the benefit of all actors in the agro-food supply chain, most importantly, the food processors, and to supplement the existing information. This review, therefore, aimed to provide a technological update on some selected non-thermal food processing methods specifically focused on their operational mechanisms, their effectiveness in preserving various kinds of foods, as revealed by their pros (merits) and cons (demerits). To compose this review paper, articles written in English and published between 2016 and 2021 (specific to those available 2021 as at the time of preparing this review) were retrieved from recognized scientific literature search engines (Science Direct, Scopus, Web of Knowledge and Google Scholar) using the key words “pulse electric field,” “pulsed light,” “ultraviolet light,” “high-pressure processing,” “non-thermal plasma,” “cold plasma,” “irradiation,” “ionizing irradiation,” and “ultrasound.” Other useful references have been added where deemed appropriate to strengthen the discussion of this synthesized update. The schematic representation of non-thermal food processing technologies specific to the overview strategy employed in composing this review is as shown in Figure 1.

## 2. Action Mechanisms/Principles Associated with the Selected Non-Thermal Food Processing Technologies

### 2.1. Physical Treatments

The physical treatments to be discussed in this section include pulse electric field, high-pressure processing, ultraviolet light, pulsed light, ultrasound as well as ionizing radiation.

#### 2.1.1. Pulsed Electric Field (PEF)

**Figure 2:**
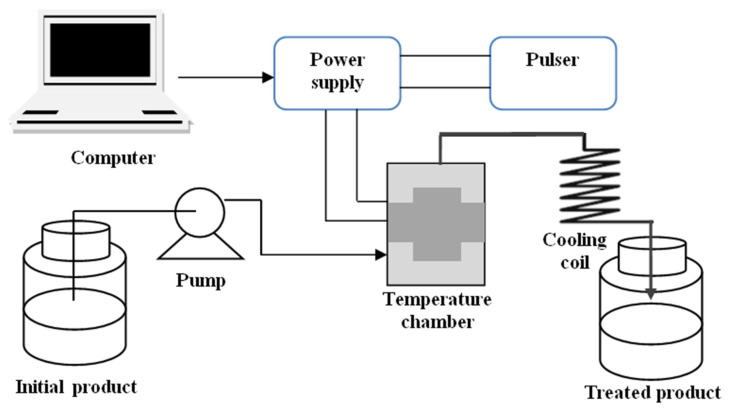
Schematic flow of PEF operation pathway (Source: Mohamed and Eissa [18]; permission to use not required).

Pulsed electric field (PEF), as it is usually called, is among the recent food processing strategies, where an electric field is utilized in place of heat. In this technique, short and high voltage pulses (usually one with an intensity that falls between ten to eighty kilovolts per centimeter and lasting for flashes of seconds) are directed via two electrodes towards the intended foodstuff [15,16,17]. Typically, a pulsed electric field scheme comprises a control and monitoring system, pulse initiator, a source of high electrical power, an assembly room for treatment, a cooling scheme that checks the heat increase, and raw and treated product compartments [8,18] (Figure 2). Usually, a negligible amount of heat, at most 40 °C, is given out in a typical PEF operation [8,19].

In principle, PEF operates with two fundamental processes, namely electroporation and electrical breakdown, which synergistically help to actualize the pathogenic destruction [9,20]. Usually, a 10–80 kV/cm intensity voltage that lasts for microseconds is applied, which results in the desired electric field responsible for the microbial inactivation. The presence of charged particles facilitates the flow of electrical energy generated to all parts of the food [2,21,22,23]. In addition, PEF can be utilized for the preservative treatment of fluid foods, such as juices [2]. To start with, electroporation results in the formation of holes on the cell wall and inner cell film of the pathogen in order to promote microbial inactivation [2,24]. When the process is combined with an electrical breakdown, the semi-permeable membrane of the pathogen is weakened. This situation momentarily results in the leakage of the cell’s cytoplasmic contents. This synergistic effect causes an irreversible death of the microbial cell [22,25]. The efficiency of a PEF treatment is based on several aspects including field strength or intensity, the rate of conductivity of the food media, its pH, the nature of pathogen involved, the temperature applied during the process, the time interval, pulse, energy applied and polarization [8,22,25,26]. PEF has mostly been applied for the treatment of foods such as apple, orange, tomato, carrot juices, apple sauce, salad dressing, pea soup, eggs [18], milk, and milk products [27]. In the fruits and vegetable industry, PEF has been applied for the enhancement of the physicochemical, rheological, and antioxidant aspects of juices [27].

As a novel technology, pulsed electric field has also shown significant control of microbes in different foodstuffs [28,29]. In a study by McAuley et al. [29], raw milk with constant flow rate of 2.4 L/min was PEF-processed at 30 kV/cm and 63 °C to achieve a microbial stability similar to that obtained by thermal pasteurization at 72 °C for 15 s. In another study, moderate intensity PEF conditions (*E* = 2.7 kV/cm and pulse width of 15–1000 μs) showed great potential in activating microbes for the processing of fruit juices [30]. Moreover, PEF proved significant in the microbial stabilization of wine compared to the action provided by SO_2_ in malolactic fermentation of wine [31]. A study by Pallar’es et al. [32] showed the significant effect of a hurdle combination of PEF and HPP in alleviating aflatoxins in grape juice. With both technologies, reduction of 14–29% was attained and an even greater reduction of 84% and 72 for aflatoxin G1 and aflatoxin B2, respectively. Such findings reveal the potential of PEF in microbial inactivation and could be of great value to not only the food processors, but also the consumers in ensuring product safety.

#### 2.1.2. High-Pressure Processing (HPP)

Over a century ago, Bert H. Hite pioneered the high-pressure processing (HPP) technology on food. HPP treatment typically involved applying pressures that reached 600 MPa [33]. However, considering works of Heinz and Buckow [34] and Mújica-Paz et al. [35], of paramount significance in a HPP process is the pressure range of 100–800 MPa and temperature as low as <20 °C applied together, with the time of exposure of the foodstuff usually seconds to minutes. Besides preventing food spoilage bacteria, unnecessary damage to the foodstuff can at the same time be prevented [36]. It is understood that the ultra-pressures in HPP systems could alter the anatomy of the bacteriological cells as well as thwart the enzymatic functions, which could result in the weakening and eventual death of the (foodborne) pathogen [37,38].

**Figure 3:**
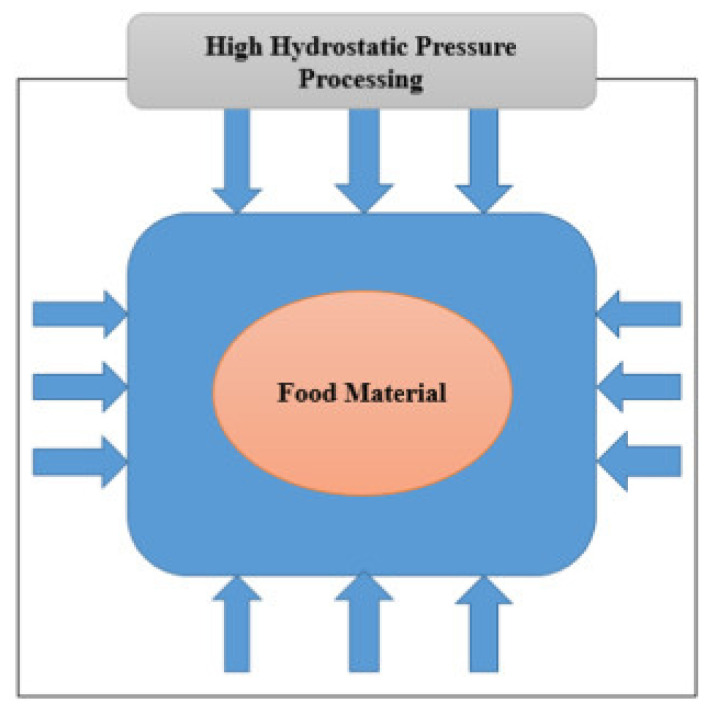
The food system under high hydrostatic pressure showing how the latter gets applied from all sides to the food material (Source: Khan et al. [36]; permission to use given by Elsevier Science).

The action of HPP on food constituents not only adheres to the Le Chatelier’s principle (structural reactions and changes favored by pressure resulting to a decrease in volume), but also to the isostatic principle (equal transmission of pressure to all parts of the liquid foods) [8,9,39,40]. Due to the compression caused by air and water, the insignificant changes in appearance [41,42] and proteins’ structure [41,43] that take place in the foodstuffs are usually irreparable. During food processing, the applied high pressure is transmitted equally to all parts of the food matrix. This is conducted through the given items in a uniform way independent of the geometry; so fast that the pressure is transferred to the medium (Figure 3) [36].

In addition to operating at normal temperature conditions, HPP can increase the foods’ shelf-life, and at the same time, prevent them from any detrimental changes on their nutritional and sensory aspects [9,17,38]. HPP has also been used for processing liquid foodstuffs, for instance, juices [2,17,38,44,45,46]. This technique is known to be applied in the treatment of foods whose water activity exceeds 0.8 [47]. Most specifically, fruits, meats, vegetables, milk and their products [48], juices, beverages, seafood, and fish [49] are known to be among the major groups processed using HPP.

HPP technology can be specifically applied in multi pulse HPP technology, involving the application of repeated short high-pressure treatment for several cycles. In a study by Szczepańska et al. [50], multi-pulsed pressure (300 MPa × 3 pulses) resulted in significant inactivation of polyphenoloxidase enzymes (57%) compared to a 31% inactivation of peroxidase enzyme by static HPP pressure (600 MPa). As also supported by Marszałek et al. [51], it is therefore quite economical to use multi-pulse at low pressures than to the conventional HPP. The significant effect of HPP against microbes has been demonstrated in different kinds of foods. In a study by Usaga et al. [52], it was shown that HPP conditions of 600 MPa for 3 min would inactivate *E. coli*, *S. enterica* and *L. monocytogenes* in beverages and juices. Additionally, ham subject to 450–600 MPa HPP conditions for 5–10 min and kept for 60 days at 4–12 °C would inactivate *L. monocytogenes* [53]. Interestingly, HPP conditions of 593.96 MPa for 233 s decreased significantly *S. aureus* and *B. cereus* in human milk, thus providing a reliable alternative to pasteurization in human milk banks [54]. Moreover, samples of fruit and vegetable smoothies subjected to HPP conditions of 630 MPa for 6 min in a temperature of 20 °C showed counts below detectable levels when kept at 25 °C for 26 days [55]. In other studies, HPP successfully combined in hurdle technology with other antimicrobial strategies such chitosan-based films (600 MPa for 8 min) against *L. monocytogenes* in ham [56] and with allyl isothiocyanate and acetic acid (250–350 MPa) against *Salmonella* in raw ground chicken meat to ensure a bactericidal effect [57,58].

#### 2.1.3. Ultrasound Technology

**Figure 4:**
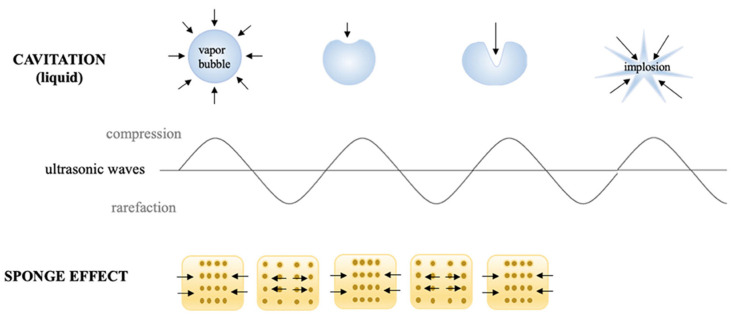
Schematic diagram showing the cavitation and sponge effect of ultrasound technology (Source: Astráin-Redín et al. [66]; permission to use not required).

As a non-thermal food protection method, ultrasound involves pressure waves with a frequency range that falls between 20 and 100 kHz [2,59]. Alternatively, it can be categorized as a minimum- or maximum-intensity sonication [4,59] or according to their frequency ranges as power ultrasound (16–100 kHz), high-frequency ultrasound (100 kHz−1 MHz) and diagnostic ultrasound (1–10 MHz) [9,60]. To deal with bacteria, the ultrasound process operates under a fundamental principle referred to as ‘sonication’ [2,9,59,61,62]. In simple terms, sonication means “to fragment by ultrasonic vibration,” which involves the creation, development, and breakdown of minute-sized foams (bubbles) inside a solution that is aqueous [9]. The bubbles generating through the restive activity of mechanical waves arise due to solicitations by intense energy. The breakdown of the bubbles creates the adverse confined pressure (approximately 1000 atm) and temperature (approximately 5000 K) conditions that cause conformational changes to the target microorganisms [41,63]. Under such localized conditions created by the sonication process, free radicals are formed from the breakdown of water molecules (Figure 4). Such free radicals (hydroxyl and hydrogen) lethally affect the microbes by destroying their cell walls, cell membranes, and liposomes, as well as genetic material. This subsequently results in cell disintegration and death [4,9,64,65]. In recent developments, a newer approach referred to as direct contact ultrasound has been implemented. This does not involve the application of fluid media (liquids or gas), but has the foodstuff placed directly on a special vessel for transduction. Under this system, the treatment effect is brought about by acoustic vibrations, which facilitate the distribution of energy and mass by the sponge effect. The sponge effect, also termed as mechanical stress, refers to the successive compressions and expansions of the food material like a sponge due to the effect of acoustic vibrations on a solid matrix. The effect mostly occurs when ultrasound is applied in dry conditions [66].

Ultrasound technology has also been used in the elimination of microbes from foods. Balthazar et al. [67] demonstrated that ultrasound conditions of 78–104 Watts in 6 min and a pulse duration of 4 s eliminated contaminant bacteria in semi-skimmed sheep milk while maintaining desired levels of lactic acid bacteria. In another study, high intensity ultrasound (0.3–3.0 kJ/cm^3^) was applied to chocolate milk and found effective against total aerobic counts by reducing 3.56 ± 0.02 logarithmic cycles as compared to conventional pasteurization (72 °C/15 s). In addition to that, the non-thermal process preserved the physicochemical, bioactive and nutritional components of the beverage [68]. In a study by Liao et al. [69], the treatment of foods with non-thermal plasma before ultrasound was found effective against *S. aureas* rather than the other way round. This was due to the fact that while non-thermal plasma provided the reactive oxygen species, ultrasound ensured they were injected into the microbe’s cells rapidly resulting to its death. It is thought that if ultrasound was applied first the microbe could have developed and enhanced oxidative response against the subsequent non-thermal plasma process. To sum it up, although many studies have proven ultrasound as a potential non-thermal technology against microbial contamination in foods, further research is required to determine how its efficiency can be improved through the combination with other technologies as hurdle. This should go hand in hand with ensuring that the physicochemical properties of foods are preserved.

#### 2.1.4. Pulsed Light (PL)

**Figure 5:**
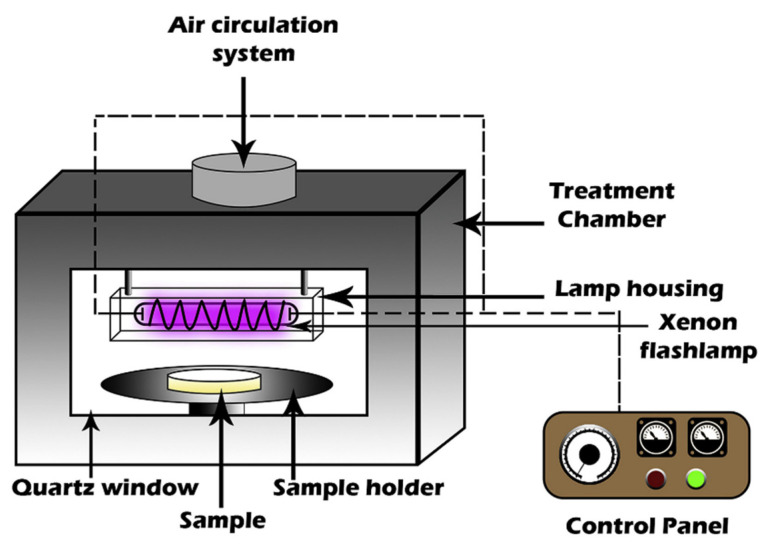
Schematic diagram of a pulsed light (PL) chamber (Source: Mahendran et al. [73]. Permission to use given by Elsevier Science).

This is among the contemporary food processing techniques in the current century that is applied in food industries. PL usually involves a comprehensive array of pulses, short but highly energized from the white light’s broadband; the latter being known to contain infrared, visible and ultraviolet light. Comparatively, PL has a thousand times strength greater than the normal UV light which is quite continuous [2,15,70,71]. Additionally, an array of highest power pulsed light can be formed in a very short period using the wavelengths of pulsed ultraviolet light (PUV) at the range of 200–1000 nm [15,72]. In the schematic diagram of a PL chamber is shown in Figure 5 [73], it should be noted that the Xenon flash lamp possesses an emission spectrum ranging from ultraviolet to infrared light. Nonetheless, of the PL band, besides being economical, UV light has been identified as the most lethal spectrum against pathogens [2,74]. In the presence of PL light, the food allergens’ conformation undergoes alterations, giving rise to the clumping of proteins. PL is also useful for microbial inactivation, affecting the microbial structures [15,72], followed by thwarting of the cytoplasmic membrane, cessation of biocatalysis and finally destruction of the genetic material [2,75]. PL can be used in the treatment of liquid foods and for those foods that have simple exterior conformations. Specific examples of foods processed by PL include fish, vegetables, fruits, and meat [73].

Similar to other non-thermal food processing technologies, pulsed light (PL) also has potential in the inactivation or elimination of microbes in foods. Romaine lettuce with thickness 0.00254–0.00762 cm subjected to direct and in-package treatment of pulsed light (1.05 J/cm^2^ s) resulted in 2.18 ± 0.25 to 2.68 ± 0.37 log CFU/g reductions of *E. coli* [76]. In a similar study, PL treatments of 8.2–12.5 J/cm^2^ on the surface of lettuce were effective by inactivating the bacterial load (*S. enteritidis*, *E. coli*, *S. aureus* and *L. monocytogenes*) [77]. Moreover, PL treatments of 0.35 J/cm^2^–3.6 J/cm^2^ exhibited significant 3–4 log reductions of *L. inocua* on sausages and about 1 log reduction in both boiled ham and chicken cold cuts. While the microbial reduction was maintained for over twelve days of 5 °C chilled storage, there were no detrimental impacts observed on the sensory attributes observed as compared to the slight deviations in high PL treatments [78]. It is worth knowing that PL can be used alongside other novel technologies as a hurdle in the inactivation of microbes on the surfaces of foods. For instance, in a study to assess the combination effect of PL and citric acid treatment against aflatoxins in peanuts, an outstanding reduction of 98.2% was achieved, a reduction that could not be obtained were the processes used singly [79].

#### 2.1.5. Ultraviolet (UV) Radiation

UV radiation (treatment) has been recently used as a non-heat technique for decontamination, improving both the shelf-life and safety of foodstuffs [9,80]. UV radiation is a form of energy considered to be non-ionizing radiation having in general germicidal properties at wavelengths in the range of 200–280 nm (usually termed UV-C). Generally, UV light falls in the range of 100 to about 400 nm and the range can be further categorized into UV-A (315–400 nm), UV-B (280–315 nm), UV-C (200–280 nm) and UV- Vacuum (100–200 nm) [81]. In principle, the UV radiation operates by destroying the genetic constituent of the pathogen to prevent division, multiplication and subsequently hinder its propagation [9,82]. Usually, different kinds of food products require different doses of UV radiation (termed as UV-inactivation dose measured in mJ/cm^2^) to inactivate different kinds of pathogens. For instance bacteria, yeast, fungus, protozoa and algae require a UV-inactivation dose of 1–10, 2–8, 20–200, 100–150 and 300–400 mJ/cm^2^, respectively, denoting that algae is the most resistant, as it requires the highest dose as compared to other pathogenic microbes [81,83]. Thus, the effectiveness of the UV radiation depends on several factors, for instance, the source and dose of the UV radiation, the duration by which the product is exposed, the nature of the foodstuff, the alignment of the apparatus, and the nature of the microbe [84].

Various studies have indicated the potential of UV light (UV-C) in preventing pathogens, with the UV light wavelength between 100–280 nm considered as germicidal. Walnuts inoculated with *Salmonella* and subjected to a UV light treatment at 8 cm for 45 s resulted to a maximum log reduction of 3.18 CFU/g. This proved an essential replacement for the non-preferred chemical and thermal treatment methods, as the physicochemical characteristics of the walnuts were not affected [85]. In another study, UV-C treatment of raw milk resulted to a 2 and 3 log decrease in the total mesophilic aerobic bacteria and yeast-mold count, respectively. Moreover, there was observed a 2–3 log reductions of the inoculated *Salmonella*, *L. monocytogenes*, *S. aureas* and *E. coli* under UV-treatment. However, to achieve a more effective reduction in bacterial load, this study indicated that UV light should not be used as a stand-alone strategy, but integrated with other technologies [86]. Additionally, in another study, a UV-C treatment of 127.2 mJ/cm^2^ for 30 s was found to be effective in reducing the bacterial load of raw salmon. This occurred due to the higher doses of UV-C, which would bring about unwanted changes in the sensory characteristics [87]. Although these findings show the significance of UV-C in microbial stabilization, it is, however, necessary that further research is conducted to determine what other technology can be used alongside UV-C as a hurdle to ensure that during microbial inactivation and that both the physicochemical and sensory properties of the foods under treatment are preserved.

#### 2.1.6. Ionizing Radiation (IOR)

Generally, ionizing radiation involves the application of gamma, electron beam/X-ray to decrease microbial contamination as well as inhibition of enzymatic activities in agro-food products [88]. Typically, the charged ions get formed when photons interact with the food molecules. These, in turn, undergo a series of changes and emerge as super-reactive free radicals, which not only react among themselves, but also with the uncharged molecules [4,36]. It is important to note that there are three technological approaches by which the mechanism of ionizing radiation operates in food processing. Firstly, there is the generation of gamma ray emission via radioactive cobalt-60 or cesium-137 with high penetrating power; secondly, X-rays that are generated at energy level not exceeding 5 MeV (high penetration) and thirdly; the emission of high energy electron beams via accelerators that operate not beyond the energy of 10 MeV (low penetration) [4,89]. In addition to that, there are three well-known concepts associated with ionizing radiation, which include radicidation, radappertization and radurization. Specifically, radicidation is said to be achieved when a lesser dosage of between 1 and 10 kGy becomes enough to destroy pathogenic/spoilage microbial entities. Radappeartization, on the other hand, signals when higher dosage above 10 kGy becomes sufficient to sterilize, for example, meat and seafood products, as well as the decontamination of seasonings and spices. However, radurization differs completely because it happens when extremely high dosage is applied, with the specific aim to extend shelf-life of a given food product. The process of irradiation of food products requires a lot of caution, explaining why the Food and Agriculture Organization (FAO) of the United Nations, the International Atomic Energy Agency (IAEA), the World Health Organization (WHO) of the United Nations, as well as the Scientific 212 Committee on Food of the European Commission (EC) have considered ionizing radiation with a dosage up to 10 kGy applied to foods as acceptable, safe and nutritionally adequate [90]. Besides, the lethality of the process can be shown when either the genetic material of the pathogen gets destroyed because of cell death owed to inhibition of DNA synthesis, or the cell splits through the generation of reactive water molecules such as hydrogen (H^+^) and hydroxyl (OH^−^) radicals. It is believed that during these processes, the physicochemical properties of foods remain unaffected. The effectiveness of ionizing radiation can be determined by the absence/presence of oxygen, its nature/types, composition of medium, food density/thickness, as well as the absorbed ionizing irradiation dose [4,91].

### 2.2. Chemical Treatments

The chemical treatments that will be discussed in this section include ozone processing/treatments, as well as cold plasma (non-thermal plasma).

#### 2.2.1. Ozone Treatment

Increasingly, ozone is becoming important in food processing, contributed by it being a GRAS (Generally Recognized as Safe) chemical with US FDA approval, as well as an antimicrobial additive for direct contact with foods [92,93]. Besides it being formed photochemically in the stratosphere, ozone has strong reactivity given its molecular structure [94,95]. In particular, the molecular configuration of ozone arises from the nature of combined sp2 and 2p2 orbitals, which brings about the dual 9-molecular orbitals representing a hybrid based on four possible structures [96,97]. Normally, temperature inversely relates to ozone solubility in water, meaning that ozone solubility decreases as temperature increases [13,98].

**Figure 6:**
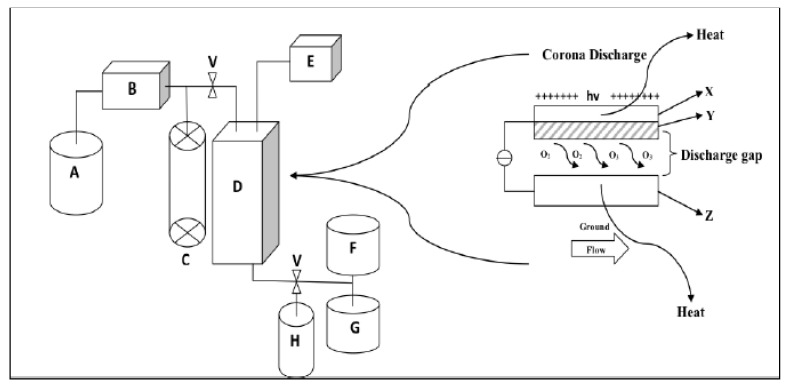
Schematic diagram for set-up of ozone generator highlighting corona discharge instrument: Key: A = Oxygen cylinder; B = Flow rate controller; C = Bubble type flow meter; D = Ozone generator; E = Transformer; F and G = Excess ozone traps (4% KI); H = Rotating vessel (90 rpm); V = Valve; X = High tension electrode (Source: Okpala, [103]; Permission to use not required).

The antimicrobial efficacy of ozone is broad in water and wastewater [13]. In the inactivation of microbes, ozone primarily acts by destroying the microbial protein structure carrying the genetic material; that is, its membrane, capsid, or envelope. This is known to be followed by a drastic reduction and cessation in the microbial ability to propagate and infect, due to further secondary integral disinfection and inactivation by ozone on the infectious pieces of genetic material resulting from the cell lysis [99]. Kim et al. [100] further clarifies that while the primary target of the destructive effect of ozone for bacteria is the cell surface; the primary site of ozone attack is known to be the double bonds of unsaturated lipids in the cell envelope. Ozone attack leads to an alteration in cell permeability that subsequently results to splitting of the microbial cell. Moreover, ozone acts by decreasing cell viability, flocculation of cellular proteins, degradation of intracellular proteins and interference with the respiratory system resulting to death due to the oxidation of sulfhydryl groups. For the case of viruses, ozone breaks down the phage coat, damage the RNA and alter the protein coat’s polypeptide chains. Ozone being highly reactive, penetrable and resulting to spontaneous decomposition has usually resulted to effective microbial inactivation in short contact times and relatively low concentrations. Generating ozone at a commercial scale within the food industry could be by one of the two procedures: (a) photochemical by passing an oxygen-containing gas through either a source of ultraviolet (UV) radiation; and (b) corona discharge (CD), which involves high-energy electrical field. Other less commercial mainstream methods to make ozone include electrolysis, radiochemical and the reaction of elemental phosphorus with water [13,101]. For instance, in the case of the electrochemical generation of ozone, there are several biocidal reactive oxygen species that can be produced. This also makes the ozone concentration to be largely dependent on the type of the electrode and the amperage [102]. A schematic diagram for set-up of ozone generator highlighting corona discharge instrument is shown in Figure 6. It can be seen that the pathway by which oxygen will be produced is controlled to ensure that the ozone is adequately generated; with provisions always made to trap the excess ozone [103].

It is important to reiterate that there are two major ways to measure the concentration of ozone in water include (a) colorimetric test kits; and (b) electronic meters. Measuring ozone concentration in food processing applications is needed to ensure disinfection. This ensures that ozone properly inactivates/kills the microbial entities [13,101]. As a result, ozone treatment has found wide applications in the food industry, like surface decontamination of fruits and vegetables, drinking water disinfection and wastewater treatment, meat, and seafood processing [13,96,104,105,106,107]. Indeed, the efficacy of ozone to reduce microbial levels applies not only to fresh produce within the domains of food preservation and packaging [108,109], but also to fumigant for insect/fungal control in grain storage [110,111].

#### 2.2.2. Cold Plasma (Non-Thermal Plasma)

Matter exists in three major states, namely: solid, liquid, and gaseous states. Interestingly, the scientific world has emerged with the fourth state, referred to as plasma [112,113]. Plasma state is attained when any gas (combined or single) is excited with high electric field strength such that it exceeds the ionization potential to change its state to the ionized form [114]. As a semi-neutrally ionized gas, cold plasma involves the combination of ions, UV photons, electrons, reactive species and charged elements [4,115,116,117]. The reactive species that constitute plasma state include those generated by oxygen (e.g., O_3_), nitrogen (e.g., NO_2_), and water (e.g., H_2_O_2_) [9,118]. Oxygen generated reactive species provide the most lethal effect against microbial cells, subjecting them to such conditions that destroy their structural components culminating in death [9,15,117,119,120,121].

**Figure 7:**
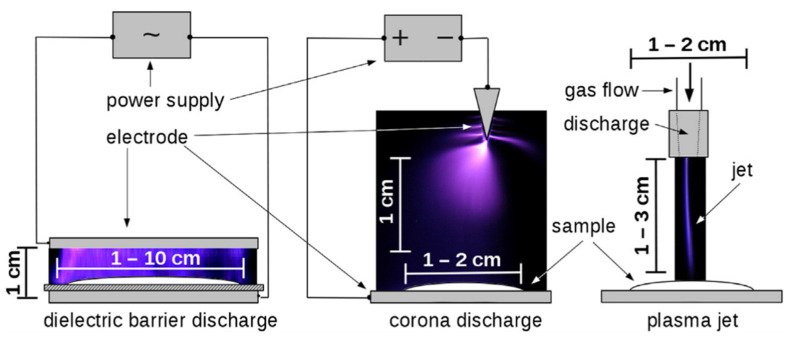
Schematic depiction of three typical electrical discharges for generating the non-thermal plasma with typical sizes indicated and discharge appearance (Source: Scholtz et al. [128]; permission to use given by Elsevier Science).

Cold plasma can act against microbes by destruction of the constituents of the microbe’s cells including their DNA [4,9,122,123]. Several methods used to develop plasma include radio frequency plasma, dielectric barrier discharges, the gliding arc discharge [113,124]; and microwave and corona discharges [9,125]. A schematic depiction of three typical electrical discharges for generating the non-thermal plasma with typical sizes indicated and discharge appearance is shown in Figure 7. Moreover, there are a number of factors that can influence the effectiveness of non-thermal plasma against microbes. There include the exposure method, management time, nature or kind of foodstuff, the properties of the intended organism’s cell membrane, the locality of the microbe, voltage used, and the kind of electrode used [9,126]. Besides identifying how cold plasma affects the molecular level of foods, the interaction of the plasma reactive species with foodstuffs are far from being elucidated, providing an opportunity for further research [127].

A number of studies have been done to determine the effect of cold plasma technology against microbes. A study by Balthazar et al. [67] on the effect of cold plasma treatment on milk showed significant results in inactivating total aerobic mesophilic bacteria and coliforms obtained through an application of 78–104 watts in 4–8 min in pulse duration of 4 s on the milk samples. In another study, corona discharge plasma jet generated by 20 kilovolts and 1.5 amperes under atmospheric pressure conditions was effective against aerobic bacteria, yeasts and molds in brown rice; maintaining the sensory and biochemical attributes [129]. A similar study investigated the potential of cold plasma against the microbiological contaminants of wheat grains whereby samples were subjected to a 0–20 min exposure to direct and indirect plasma in a contained reactor dielectric barrier discharge system. The process was found effective against the pathogens inoculated on the surface of the wheat grain [130]. Moreover, other studies on the antimicrobial effectiveness of cold plasma technology in packaged ready to eat foods such as in chicken meat [131] and in beef [132] have indicated that apart from the controlling effect against microbes such as *E. coli*, *S. typhi* and *L. monocytogenes;* cold plasma had no detrimental effects on the sensory and biochemical aspects of the packaged foods.

## 3. Merits Associated with the Selected Non-Thermal Food Processing Techniques

The notion that non-thermal food processing techniques would exhibit less or no changes in the sensory attributes of food does not necessarily apply across all non-thermal food processes. Additionally, some of them are efficient with respect to energy consumption and time utilization, and others provide assurance for the freshness, safety improvement, and an increased shelf-life of foodstuffs compared to the conventional methods that utilize heat [133]. In the following section, the advantages/merits associated with the selected non-thermal food processing covered in this study have been discussed.

### 3.1. Physical Treatments

#### 3.1.1. Pulsed Electric Field (PEF)

PEF, which exhibits a higher application in the processing of liquid foodstuffs compared to solids [22], is known to retain nutrients (heat-sensitive foods), sensory characteristics, promote durability and ensure the foodstuffs are safe. Besides utilizing as low amounts of energy as possible, PEF is considered an environmentally friendly and highly energy-efficient technique, different from the conventional heat processing methods [22,134]. Bhattacharjee, Saxena, and Dutta [8] reported that PEF, alongside its bacteriological effects against microbes, performed better compared to other non-thermal methods in processing watermelon juice, with the retention of high amounts of anthocyanins after treatment. Furthermore, Hernández-Hernández, Moreno-Vilet, Villanueva-Rodríguez [4], and Barba et al. [26] demonstrated PEF with several other benefits compared to the traditional (heat) processing methods for juice treatment, which included decreases in energy costs, processing time, and degradative effects of heat-sensitive food components, as well as facilitating increased transmission of mass.

Bhat et al. [135] showed that PEF enhanced the salty taste in food products by manipulating the dispersion of salt and distribution of sodium, subsequently improving chewability. This suggested that PEF is useful in the production of better lowered-sodium meats. A recent investigation by Alles et al. [136] into the bio-refinery of insects with PEF showed low levels of PEF (<3 kV/cm; 5 kJ/kg) would not result in insect mortality, but would bring about reductions in oil droplet size in the insect biomass. The application of PEF can have other such effects as modulating mineral contents, extracting oils and assisting in the drying processes in foods. Another recent study by Shorstkii et al. [137] investigated the optimization of PEF assisted drying process of black soldier fly larvae. These researchers showed that with respective drying temperature and specific PEF energy input ranges between 81 and 84 °C, as well as 11.2 and 13.1 kJ/kg, a specific an optimal processing window for larvae drying can emerge. From this, the PEF energy affected consumption of drying energy much less, but more by drying temperature. Moreover, the use of PEF can enhance the extraction of bioactive compounds from food by-products. Huge amounts of total soluble solids content were obtained after a PEF treatment of 10 and 15 kJ/kg of cranberry bush purée, denoting significant improvement in extractability of bioactive compounds. Additionally, there was an increased retention of bioactive compounds; total phenolic content of ~10–14% and total flavonoid content of ~6–8% after PEF treatment of 3 kV/cm; 5–15 kJ/kg [138].

#### 3.1.2. High-Pressure Processing (HPP)

HPP technology is applied in processing foodstuffs that are either solid or liquid [26]. Since the technology involves less-to-no utilization of food preserving agents [4,139], relatively low amounts of energy, and can reuse the transmission and pressurization fluid (water) with zero-emission of wastes [47,140,141], it can be considered as one among the eco-friendly types of non-thermal food processing techniques compared to thermal pasteurization [142]. Moreover, HPP can retain the taste of food, its nutrient composition and elongate its shelf-life. Thus, the spoilage rate can be decreased, which can help raise the economic value of the food commodity [15,143,144]. A study by Rode and Rotabakk [145] demonstrated how HPP extended the shelf-life of codfish to at least forty-nine days. HPP can help in the shucking of aquatic products, maintaining the integrity of the flesh with a meat recovery rate of up to 100% [144,146].

The vitamin C content in an HPP-treated fruit juice resembled that of refrigerated fresh juice. This suggested the HPP process negligibly influenced the bioactive elements of foods [8]. However, health-promoting features of foods, which are of greater importance, through HPP could have its bioavailability augmented; not only the phytochemical, but also the food trace elements. Furthermore, HPP could help retain the good fats, lower salt consumption, and decrease the allergen development and toxin generation capacity of foods [26,147]. HPP has been shown to possess high ability to extract or recover high-added value antioxidant bioactive compounds from food materials, for example winery wastes and by products. For instance, HPP has shown significant potential to significantly increase the total as well as individual anthocyanin content, compared with the conventional extraction methods [26]. Additionally, when matched against the conventional methods that employ the use of heat, the hydrostatic pressure effects exerted by HPP have been considered independent of the product magnitude, and geometrical alignment [147]. Once the operational pressure has been attained, however, there is usually no extra energy needed to uphold the pressure. Compared to the conventional heat-utilizing technologies, HPP does not require supplementary energy to cool the food product beyond the estimated treatment period [47].

#### 3.1.3. Ultrasound Technology

The advantages of ultrasound technology include the use of a highly reduced treatment time for handling foods, the use of a minimal amount of energy, and greater material input and output [4,148]. Moreover, it has attracted more preference, as it is considered safe and environmentally friendly [15]. Additionally, the technique is considered quite feasible, as it is simple and economically cheap compared to the conventional heat processing methods [9,149]. In a study by Guimarães et al. [150], it was interestingly found that ultrasound resulted in shorter processing time and increased probiotic viability when applied on probiotic-made dairy products. In the dairy products with low lactose content, ultrasound at high intensity brought about higher oligosaccharides concentration, lower acetic and propionic acid content (less undesirable taste) and reduced constituent ingredients (less-to-no need of prebiotic addition or beta-galactosidase inclusion). It was also revealed that, in dairy products such as cheese, high-intensity ultrasound could decrease ripening time, but would accelerate proteolysis, which would bring about better nutritional (bioactive peptides), sensorial and textural properties. Ojha et al. [151] reviewed ultrasound technology for food fermentation applications and showed that at low frequency between 20–50 kHz, both cell permeability and mass transfer could be improved to result to better process efficiency and production rates. Ultrasound at low frequency would also stimulate the prevalence of probiotics (living “friendly” bacteria largely popular as food supplement). The mechanism is such that there would be accelerated lactose hydrolysis and transgalactosylation of bifidobacteria in milk, and at the same time, decline in fermentation time by up to 30 min, and all these would largely depend on the probiotic strain. Additionally, the authors showed ultrasound could be used to eliminate microbial entities that would hinder the food fermentation processes.

#### 3.1.4. Pulsed Light (PL)

PL serves as a rapid disinfection food processing technology. In addition, it exhibits much less damage to the nutritional content of foodstuffs that it has been applied to [90]. PL is also shown to ensure microbial inactivation while at the same time retaining the foodstuff’s sensory characteristic with fewer losses in terms of quality [152]. The technology boasts a huge advantage compared to UV radiation by exhibiting an outstandingly short time energy transmission [152,153]. Furthermore, besides the fact that PL exhibits a substantial reduction of bacteria in an exceptionally short time; it has huge adaptability, and is eco-friendly [152,154]. Consequently, after PL application, the threat due to food-inherent disease-causing microorganisms is decreased; the shelf-life of foods increased as well as a promised enhanced economic return especially during the transportation period [152,155]. In addition to that, PL has demonstrated promising results in the prevention of contamination of packaged products; the treatment is known to be applied even when the food is within the packages [156].

#### 3.1.5. Ultraviolet (UV) Radiation

Research has shown that when UV radiation is used for the processing of fruit juices (e.g., watermelon juice), its nutrient contents are better preserved. It has mild effects on the amounts of phenolic compounds, Vitamin C, lycopene, and antioxidant capabilities [8]. Additionally, the lethality effects of UV radiation against microbes are higher compared to the conventional chemical agents, for example, hydrogen peroxide and chlorine [9,80]. Moreover, UV radiation is easy to utilize (user friendly) and cost-efficient [8,157], has minimal effects on the quality of foods as it enhances sensory features such as taste for certain foods, prevents recontamination as it can be applied in already packed food products, is environmentally friendly, can be used not only for liquid foods, but also for solid ones, its processing time described as shorter, and it also exhibits outstanding permeation capabilities to foodstuffs [4,36].

#### 3.1.6. Ionizing Irradiation (IOR)

It is among the non-thermal preservation methods with minimum effect on the quality, taste, appearance, and texture of foods. Ionizing radiation acceptability elevates as the consumer desire for minimally processed and yet safe food increases [158]. The effectiveness of ionizing radiation is not only against destroying microbial entities and inhibiting pathogenic/spoilage bacteria; but also inhibiting insects, mites, and pests [89]. The application of ionizing radiation has been shown as an alternative technique to detoxify aflatoxin present in foodstuffs. The ionizing radiation has been shown to help maintain the freshness in freshly consumed food products such as salmon without significantly affecting their sensory qualities as color, odor, taste, and texture. The ionizing radiation has been shown capable of destroying the pathogenic and spoilage microorganisms for example *Listeria monocytogenes* and *Vibrio parahaemolyticus* [159].

The processing time of ionizing radiation is reasonably less, considered as eco-friendly as well as leaving no chemicals/residue [90]. Ionizing radiation technology is currently chosen as an alternative to chemicals and heat application when it comes to pathogen control. Ionizing radiation is not only capable of breaking the phosphodiester and hydrogen bonds in DNA strands, causing inhibition of microbial growth, but also able to reach the irradiated pores that cannot be achieved by other sterilization methods [160,161]. Besides, ionizing radiation technology increases shelf life without altering the texture and firmness of foods like mushrooms and cheese [162]. Besides, the dosage of ionizing irradiation applied for food preservation is usually lower and of no harm to humans upon consumption of irradiated foods [163].

### 3.2. Chemical Treatments

#### 3.2.1. Ozone Treatment

Ozone is generally recognized as safe (GRAS) and approved as an antimicrobial agent of direct application to foods [13,101,164]. Ozone cleans and disinfects better than chlorine because of the latter’s relatively low inactivation rate owed to concentration limitations posed by regulations [13]. The electronic method for dissolved ozone operation measures the sample in real-time, which allows control of (ozone) generation, as well as determination of (dissolved) levels [13,101,103]. The lower energy overall energy consumption of ozone is worth mentioning as a strong merit [13].

Researchers consider the higher weight per cent ozone product as an economic advantage. This is due to the fact that the equipment footprint is smaller given the higher ozone’s solubility in water. This means that smaller contractors and pumps would involve less energy cost as well as space for ozone facility [13]. However, excess ozone rapidly auto-decomposes into oxygen, leaving no residue in the food product [13,101,103]. The high efficacy of ozone against a wide range of such microbial entities like bacteria, fungi, protozoa, and viruses has been demonstrated [13,101,165]. Ozone has very great biocidal activity at reduced contact times [13,164]. Another advantage is the possibility to use ozone in the gaseous or liquid form (gaseous ozone or ozonated water in the food industry) [13,101,103].

#### 3.2.2. Cold Plasma (Non-Thermal Plasma)

All kinds of microbes are said to be inactivated by cold plasma technology, including viruses, fungi, and bacteria [117,166]. The utilization of non-thermal plasma as a non-heat food treatment method is based on the fact that it results in the retention of the original taste of foodstuffs as well as their nutrient contents [113]. When compared to thermal technologies, cold plasma involves the utilization of quite a low temperature [15,167]. Since the cold plasma process can be carried out at cold/room temperatures [90], it results in minimized losses to the foodstuffs in terms of quality, most especially retaining the heat-sensitive elements [9,126]. Similarly, cold plasma differs from conventional methods by using a lesser amount of water and is also regarded as cost-effective (reduced expenditure due to the use of natural gas and electricity) [4,168].

Cold plasma can serve for in-package sterilization [90]. It has continually been referred to as an eco-friendly technique since, besides having minimal changes on the food matrix, its application does not result to the generation of toxic residuals/wastes [90,169,170]. Moreover, the technology has similarly been described as lowering the immune-reactivity of foods [15]. Furthermore, the technology is used for the treatment of foodstuffs that have pathogens situated on the outer parts, the treatment effect reported to be reaching all sides of the food matrix. It can also be applied for surface decontamination during the period of packing foods. Additionally, for minimal surface treatment of fresh meats and greens, cold plasma is thought to be the most suitable option [171]. With respect to seafood, cold plasma has been recognized a promising non-thermal preservation method because of its high inhibition efficacy to tackle various microbial entities [172].

## 4. Demerits Associated with the Selected Non-Thermal Food Processing Techniques

In the following section, the demerits associated with the selected non-thermal food processing technologies covered in this study have been discussed.

### 4.1. Physical Treatments

#### 4.1.1. Pulsed Electric Field (PEF)

The disadvantages associated with PEF include: huge start-up costs as one of the main obstacles to the application of PEF on large capacity. The various kinds of equipment required for its initial set-up are very expensive, their price ranging from 250,000–2,000,000 USD [22,134]. Therefore, to achieve complete industrial applications, there is a need to improve the technique and to upgrade the capacity of the equipment [4,173]. There could also be the failure for effective processing. This could be due to the fact that some bacteria cells (vegetative and spore) have developed resistance against the pulsed electric field method. This might eventually result in a public health risk [9,20]. PEF has been reported as not yet effective in treating solid foodstuffs, compared to partial solids or liquid foods such as juices and boiled meats [4,174]. Besides, it has not been easy to successfully scale-up PEF given the fact that the technology is quite sophisticated and only a handful of studies are currently available regarding its various industrial aspects [175].

#### 4.1.2. High-Pressure Processing (HPP)

HPP is considered not suitable to be applied in dehydrated and porous foodstuffs [2]. This is because while such foods demand to be maintained in their dry conditions, the use of water is, however, indispensable during the HPP process [46]. Besides, HPP treated foodstuffs have to be kept in cold/refrigerated conditions. This is because pressure applications alongside such temperatures are effective in inactivating vegetative cells of microbes compared to when pressure is used alone. Although this makes the HPP method appear efficient, it might turn out to be uneconomical and laborious [46]. Moreover, to the best of our knowledge, only plastic materials appear the best fit as packing materials for HPP products, as the process requires packaging materials that can be compressed to some degree, at least about 15% [46], which appears to be a setback given that it prevents the use of multiple types of packaging materials. HPP is also reported to adversely upset milk and dairy food product components; for instance, reducing the casein micelle magnitude, raising the free fatty acids level as well as altering the natural qualities of whey [176]. Like many other non-thermal processes, HPP is significantly hampered by huge initial charges. In particular, like PEF technology, high equipment costs (even over $7000) should be considered [102]. This is, however, thought to be minimized by the operational costs, which are seemingly lower [48]. Moreover, the complex and large nature of the facility would require appropriate skill and space to effectively operate.

#### 4.1.3. Ultrasound Technology

During ultrasound application in liquid foodstuffs, the cavitation process is reported to generate free radicals that may be the starting point of the decline of food product quality [9,177]. These subsequently lead to oxidation of lipids, denaturation of proteins, and degradation of ascorbic acids with associated detrimental changes in sensory attributes [178]. Ultrasound technique is shown to produce detrimental effects on the characteristics of foodstuffs such as the sensory parameters as well as nutrient composition [4,179].

Besides, at ambient temperature and pressure, high power ultrasound wave (20 kHz) has been shown to produce low inactivation of some micro-organisms, especially *Listeria monocytogenes*, which can be ameliorated either by increasing the power of sonication or by an increase in pressure (manosonication) [10]. There still appears to be limitations regarding the industrial scale of ultrasound. This has to do with the risks it poses to the operators/workers. There is evidence to show that ultrasound technology can pose occupational risks to operators. Magnavita and Fileni [180] showed that contact ultrasound would pose greater hazards compared to exposure to airborne ultrasound clearly because the air transmits much less than one percent of this kind of energy.

#### 4.1.4. Pulsed Light (PL)

Similar to UV technology, the pulsed light comes with huge capital/costs in order to achieve successful investment [152]. Pulsed light has been shown as not suitable for application in foods that are opaque and irregularly shaped, as they can be potential habitats for bacteria proliferation [152]. Moreover, extended periods of treatment in PL can result in a “heating effect” upon food products and in the end affect the effectiveness of the bacterial destruction [152,181].

#### 4.1.5. Ultraviolet (UV) Radiation

The major setback that UV irradiation has faced for quite an extended period is the lack of complete recognition and acceptability from the consumers. Unfortunately, many people are still skeptical, especially on whether foodstuffs that have been handled by UV radiation are harmless [4,36]. Consumers seem to be worried about the fact that UV radiation might be leading to radioactive materials in foods, which may subject them to serious health issues [9,82]. Indeed, UV-C light could represent risks for humans if they are exposed to it. For instance, UVC radiation can cause severe skin burns and eye injuries (photokeratitis) [182]. This UV-C limitation would suggest that its continuous exposure to materials or processing surfaces could affect their chemistry and properties.

In addition, the UV radiation could also cause isomerization and oxidation of lycopene especially with increased radiation concentrations and contact times [8]. Another limitation is that the effect of UV-C light application on liquid foods is affected by their turbidity [102]. The time-consuming and laborious task to ship ready-made foodstuffs to irradiation plants limits the applicability of UV radiation in the treatment of food products after packaging [9,40]. When applied to foodstuffs with indefinite shape and structure, UV radiation would be quite ineffective given its low penetration capacity. This would, however, improve if UV radiation is combined with other non-thermal processes [9]. Additionally, huge investment requirements are also a limiting factor for achieving the complete feasibility of the UV radiation process [4].

#### 4.1.6. Ionizing Irradiation (IOR)

One of the disadvantages of IOR is that when the ionizing radiation dose is too high, the functional as well as the sensory properties of foods such as color and odor could be affected. This implies that apart from depending on the degree of irradiation, the required dosage to be applied to foods would be dependent on product parameters like geometry/smoothness, speed of transmission, the density of the product, as well as the load patterns [159]. Besides, ionizing irradiation can speed-up the auto-oxidation of lipids, producing hydroperoxides and off-flavors with no increased threat due to mycotoxin formation. The method is also found that it could have a direct effect caused by oxygen-entered reactive radicals from water radiolysis, where higher dosage would change both flavor and aroma of food product [163]. Ionizing radiation could also become harmful and emerge as a threat to processors and workers upon excess accumulation as a result of constant exposure to irradiation [183]. The huge capital/investment requirements associated with the purchase of ionizing radiation facility is also another disadvantage of the IOR technology.

### 4.2. Chemical Treatments

#### 4.2.1. Ozone Treatment

Ozone facilities especially those of commercial scale involves high initial capital and maintenance costs [13,184]. Usually very toxic when inhaled, ozone is corrosive if used above 4 ppm [13,184]; making it a requirement to effective monitoring especially in indoor applications [13,184]. Since the half-life of ozone is very short [13,164] it has been shown to lack stable residual that limits its online testing efficacy [13,164].

At the large commercial scale levels, an ozone process facility/system would require skilled trained operators given the complexity as well as occupational safety precautions, especially industrial ozone processes [13]. The available concentrations of ozone, like the domestic types (as well as some of the commercial types) are usually fixed [101,103,107] and while this can pose challenges, it will also require a lot of caution during handling. Another drawback is the manner in which ozone reacts with organic matter, which could be limiting either the antimicrobial action or the efficacy of the treatment [13].

#### 4.2.2. Cold plasma (Non-Thermal Plasma)

There are a number of suppositions that free radicals are generated by cold plasma. The free radicals are usually very quick to undergo oxidation and may result in lipid deterioration and destruction of inherent antioxidants, leaving the food with an undesirable taste and aroma [113,185,186,187,188,189]. Foods subject to cold plasma are believed to have challenges of attaining a 5-log decrease of bacteria cells or spores when used singly. As a result, it has to be used in combination with pressure and/or temperature [2,62]. The high capital investment required to set up plasma generating equipment, and challenges associated with understanding the plasma chemistry is among key demerits of this non-thermal technology [1,86]. Additionally, cold plasma experiences a lack of optimized process and product factors; limiting effective scale-up of the process from lab or pilot to industrialized stage to ensure its cost-effectiveness [4,174,189].

Cold plasma could speed up the oxidation rate of lipids in dairy products, adversely impacting their sensory attributes [169]. There are difficulties encountered, especially in treating foods with no definite shape and structure, probably because the plasma effect is unable to reach the food matrix [169,190]. This might leave some bacteria untreated due to their attachment to many localities [169,188]. Further challenges include the fact cold plasma technique is still at its lowest level (laboratory or pilot scale) [169,191,192]. The full feasibility of the non-thermal cold plasma technique to food products still remains unclear, largely due to the lack of clearly stipulated functional settings given the presence of only a handful of studies regarding its effects on product quality [169,190,192].

## 5. Final Considerations

The action mechanism of the selected non-thermal technologies we have reviewed herein appears driven by a wide range of energy sources, for instance, electrical fields, light, and hydrostatic pressure. These techniques have proven their capacity in keeping foodstuffs near an original fresh state, which is much desired by consumers, different from the detrimental effects caused by many of the conventional heat application techniques. Some selected non-thermal technologies produce minimal effects on the nutritional composition of foodstuffs and promote the bioavailability of some bioactive components of foods.

Moreover, the design of equipment of large-scale application, formulation of rules and regulations governing the safety of foodstuffs being processed, expanding the process mechanisms of each technique, as well as raising awareness through consumer education, are among several essentials of such non-thermal technologies that makes their adoption at industrial scale challenging [133]. Since the majority of the novel non-thermal technologies are still utilized at a small scale (lab/pilot level), their scaling-up to an industrial level might prove expensive and therefore, the huge investment costs requires consideration for sustainable implementation and utilization [8]. Therefore, further design of such non-thermal technologies facility requires improvement, which could make the eventual process mechanisms better and cost-effective [22].

Consumers will continue to demand for nutritionally fresh foodstuffs, and for this reason, the onus remains on the food industry to push towards fully embracing non-thermal food processing technologies, especially those discussed in this review. To the consumers, this synthesis provides some relevant information that could help them in deciding their preferred foodstuffs to consume based on the non-thermal processing methods. Overall, this current synthesis can serve as a quick reference for food processors who intend to employ one or more of these selected technologies.

## 6. Future Prospects

As a way forward and into the future, it is important for food industries to fully understand the respective action mechanisms, as well as pros (merits) and cons (demerits) of non-thermal food technologies, prior to and even during their implementation. Streamlining the process mechanisms of each technique and consumer education about the strengths and prospects of non-thermal technologies could help to raise awareness, prior to considerations on how to amend their designs if their cost-effectiveness and scale-up capacity for industrial-level applications are to be improved.

Essentially, the implementation as well as selection of innovative non-thermal technologies within the food industry should involve a deep evaluation of the processing line via hazard analysis and critical control points (HACCP) methodology. Such deep evaluation would require combined efforts of HACCP and quality assurance control points (QACP), which can help to enhance and sustain the improved food hygiene, quality and safety processes [193]. In addition, future studies should be directed to perform cost comparisons of the selected non-thermal food processing technologies. Such cost comparisons can help the food industries, as well as their respective stakeholders to select the appropriate non-thermal technology that meets their food production requirements based on their capacities and operational needs.

Additionally, the developing of a (hurdle-like) non-thermal technology that combines a number of processing methods, designing the intended equipment particularly for large scale application, as well as formulating the rules/regulations governing the intended foodstuff safety when using these technologies, should be among the future priorities for the food industry and its stakeholders. It is important to reiterate here that when a target food industry that operates at either small-, medium- or large-scale desires to implement a specific non-thermal food processing technology, the prerequisites already prescribed by the manufacturers should be adhered to despite the variations in facilities/equipment, operational/production scales, intended food product(s), factors of production, as well as consumer targets.

## Data Availability

Not applicable.
